# *Drosophila* VAMP7 regulates Wingless intracellular trafficking

**DOI:** 10.1371/journal.pone.0186938

**Published:** 2017-10-24

**Authors:** Han Gao, Fang He, Xinhua Lin, Yihui Wu

**Affiliations:** 1 School of Life Sciences, University of Science and Technology of China, Hefei, China; 2 State Key Laboratory of Membrane Biology, Institute of Zoology, Chinese Academy of Sciences, Beijing, China; 3 University of Chinese Academy of Sciences, Beijing, China; 4 State Key Laboratory of Genetic Engineering, Institute of Genetics, Collaborative Innovation Center of Genetics and Development, School of Life Sciences, Fudan University, Shanghai, China; 5 Division of Developmental Biology, Cincinnati Children’s Hospital Medical Center, Cincinnati, OH, United States of America; 6 State Key Laboratory of Stem Cell and Reproductive Biology, Institute of Zoology, Chinese Academy of Sciences, Beijing, China; Simon Fraser University, CANADA

## Abstract

*Drosophila* Wingless (Wg) is a morphogen that determines cell fate during development. Previous studies have shown that endocytic pathways regulate Wg trafficking and signaling. Here, we showed that loss of *vamp7*, a gene required for vesicle fusion, dramatically increased Wg levels and decreased Wg signaling. Interestingly, we found that levels of Dally-like (Dlp), a glypican that can interact with Wg to suppress Wg signaling at the dorsoventral boundary of the Drosophila wing, were also increased in *vamp7* mutant cells. Moreover, Wg puncta in Rab4-dependent recycling endosomes were Dlp positive. We hypothesize that VAMP7 is required for Wg intracellular trafficking and the accumulation of Wg in Rab4-dependent recycling endosomes might affect Wg signaling.

## Introduction

Wnts are evolutionarily conserved glycoproteins that regulate patterning and growth at multiple steps throughout development [1, 2]. Abnormal Wnt signaling is involved in many human diseases, such as developmental disorders and cancers [1]. Wingless (Wg), the *Drosophila* homolog of vertebrate Wnt-1, activates signaling pathways in its receiving cells as a short-range organizer and a long-range morphogen [3, 4]. Over the last three decades, the mechanisms regulating Wnt secretion and trafficking have received considerable attention. However, these mechanisms remain incompletely understood.

Wg trafficking is tightly controlled by numerous players [5]. In the producing cells, Wg is palmitoylated by Porcupine in the endoplasmic reticulum [6–9] and escorted to the plasma membrane (PM) by Wntless [10–12], which is then recycled back to the Golgi by the retromer complex [13–17]. Several hypotheses have been proposed for how Wg is released for trafficking. Previous studies have suggested that Wg may be loaded into endosome-derived exosomes for export after endocytosis [18–21]. Another model proposes that Wg could be first internalized from the apical surface and then released from the basolateral side after transcytosis [22]. Both models indicate that the endocytic pathway is important for Wg trafficking.

Current knowledge supports the Wg endocytic pathway model: when being internalized into cells, Wg is packaged into early endosomes facilitated by Rab5 GTPase [23]. Shortly afterward, part of Wg is recycled back to the PM through Rab4-dependent rapidly recycling endosomes, while another part of Wg is trafficked further inward into multivesicular bodies (MVBs) [23]. From MVBs, the molecules can be either recycled back to the cell surface through Rab11-dependent slow recycling endosomes or delivered to the lysosome. However, the importance of each pathway is not clear, especially the functional significance of the Rab4-mediated pathway.

In addition to Rabs, the SNARE (soluble N-ethylmaleimide-sensitive factor attachment receptor) proteins are also known to play a critical role in vesicle fusion [24]. During fusion, SNARE proteins in opposing membranes specifically recognize each other and then assemble into a stable complex [25]. Deletion of or reduction in SNARE proteins can cause a significant impairment in vesicle transport. SNARE proteins may also be involved in Wg transport. For example, Ykt6 was reported to influence Wg secretion [18]. The SNARE protein VAMP7 (vesicle-associated membrane protein 7) has been suggested to mediate general post-Golgi trafficking events [26, 27]. Recent reports have suggested that *Drosophila vamp7* is the single homolog of mammalian *vamp7* and *vamp8* [28–30]. VAMP7 and VAMP8 are both involved in the fusion of late endocytic organelles [26], but whether VAMP7 regulates Wg trafficking has yet to be elucidated.

In addition to intracellular trafficking, Wg transportation and signaling activity are also regulated by membrane-associated proteins and secreted proteins in the extracellular space [31]. Dally-like (Dlp) is a GPI-anchored glypican containing glycosaminoglycan (GAG) chains in its core protein. Dlp not only competes with the Wg receptors to suppress high levels of Wg signaling [32] but also interacts genetically with Notum [33–35]. As reported recently, Notum is capable of deacylating Wg as a secreted carboxylesterase in the extracellular space for negative feedback of Wg signaling [36, 37]. Therefore, Dlp is thought to help bring Wg to Notum to negatively regulate Wg signaling. However, the detailed mechanism is not well understood.

In this report, we attempted to characterize the roles of VAMP7 in Wg trafficking and signaling. We found that loss of VAMP7 altered Wg distribution and reduced Wg signaling. We further investigated why Wg signaling was affected and where the transport of Wg was interrupted. We showed that the increase of Dlp may suppress Wg signaling when both Dlp and Wg accumulated in Rab4-dependent recycling endosomes.

## Materials and methods

### *Drosophila* strains

For the *vamp7* mutant allele, a P-element insertion line [y w; P[38]Vamp7^G7738^/CyO (BL28488)] that was previously reported by Takats was used [28]. The P-element insertion is 24 nucleotides downstream of the translation start site in the coding sequence, and this mutant allele is pupal lethal [28]. The *vamp7* mutant allele was combined with *FRTG13* to generate *vamp7* mutant clones. The *vamp7* mutant allele was also combined with *UAS-Rab11-GFP* (BL8506) or *ci*^*Gal4*^ to label the slow recycling endosomes. The *ap*^*Gal4*^ *UAS-Dcr*^*2*^, *ci*^*Gal4*^, *en*^*Gal4*^ *UAS-Dcr*^*2*^, *Wg*^*LacZ*^, *YRab4 [39]*, *UAS-Rab11-GFP* (BL8506), *UAS—GFP*/CyO (BL42705), *UAS-Rab7-GFP*/CyO (BL42705) and *UAS-dlp*^*RNAi*^ (BL34089) stocks and constructs are described in FlyBase and were obtained from the Bloomington Stock Center. The *UAS-vamp7*^*RNAi*^ transgenic line was generated in this study.

### Generation of *vamp7* mutant clones

The *vamp7* mutant clones were produced by the FLP-FRT method [40]. Loss-of-function clones in the wing disc were induced in *yw hsp70-flp/+* or *Y; FRT*^*G13*^ *ubiquitin-GFP/FRT*^*G13*^ *vamp7* animals by heat shock 24 h and 48 h after crossing at 37°C for 90 min, as described previously [40]. The *vamp7* mutant clones were characterized by the absence of GFP.

### Generation of the shRNA allele

To perform RNA interference (RNAi) in the imaginal discs, we generated the shRNA allele of *vamp7*. The shRNA against *vamp7* was designed using DSIR (http://biodev.extra.cea.fr/DSIR/). The top and bottom strands were synthesized, annealed at 95°C for 5 min and cloned into the pWALIUM20 vector as described [41]. The sequence selected was as follows:

5ʹ-GCGAGAACAAACTGGTCTACA-3ʹ.

### Antibodies and immunofluorescence

Fixation of imaginal discs and antibody staining were performed using standard procedures [42]. The primary antibodies included mouse anti-Wg (1:4, Iowa Developmental Studies Hybridoma Bank (DSHB Cat# 4d4, RRID:AB_528512), rat anti-Ci (1:10, DSHB Cat# 2A1, RRID:AB_2109711), mouse anti-Engrailed (1:10, DSHB Cat# 4D9 anti-engrailed/invected, RRID:AB_528224), mouse anti-Dlp (1:10, DSHB Cat# Dally-like (13G8), RRID:AB_528191) [43], which were monoclonal antibodies. In addition, the following polyclonal primary antibodies were also used: guinea pig anti-Wg (1:200 made by our lab) [44], guinea pig anti-Sens (1:500 obtained from Bellen HJ’s lab) [45], rabbit anti-Wg (1:200 obtained from Cumberledge S’s lab) [46], rabbit anti-β-Galactosidase (1:1000, MP Biomedicals Cat# 08559762, RRID:AB_2335286), chicken anti-beta Galactosidase (1:1000, Abcam Cat# ab9361, RRID:AB_307210), rabbit anti-Rab5 (1:500, Abcam Cat# ab31261, RRID:AB_882240), and rabbit anti-Rab4 (1:140, Abcam Cat# ab78970, RRID:AB_2042753), rabbit anti-LAMP1 (1:500, Abcam Cat# ab30687, RRID:AB_775973). The fluorescent-conjugated secondary antibodies (donkey Anti-rabbit IgG, donkey anti-mouse IgG, donkey anti-guinea pig IgG and donkey anti-rat IgG, donkey anti-chicken IgM, which were conjugated with Alexa Fluor 488, Cy3, or Cy5) were obtained from Jackson Immuno Research Laboratories, Inc. Images were obtained using a Zeiss LSM 780 laser-scanning microscope (Carl Zeiss).

### Intensity plot and data analysis

Intensity plotting lines were derived from data of average fluorescence intensity in the rectangular regions marked by with same color or the whole images. Each pair of rectangles for comparative analysis was in equal shape and size, and the data were collected along the direction of the arrows. The number of fluorescence positive puncta was counted in the regions of five different wing discs. The proportions of endosomes marker and Wg double positive puncta in total Wg puncta were also calculated, and their percentages were summarized. Multiple t tests analyzing of GraphPad Prism 7 were used to calculate P value. In all figures, n.s., not statistically significant, *, p<0.01, **, p<0.001.

## Results

### VAMP7 contributes to Wg distribution

**Fig 1:**
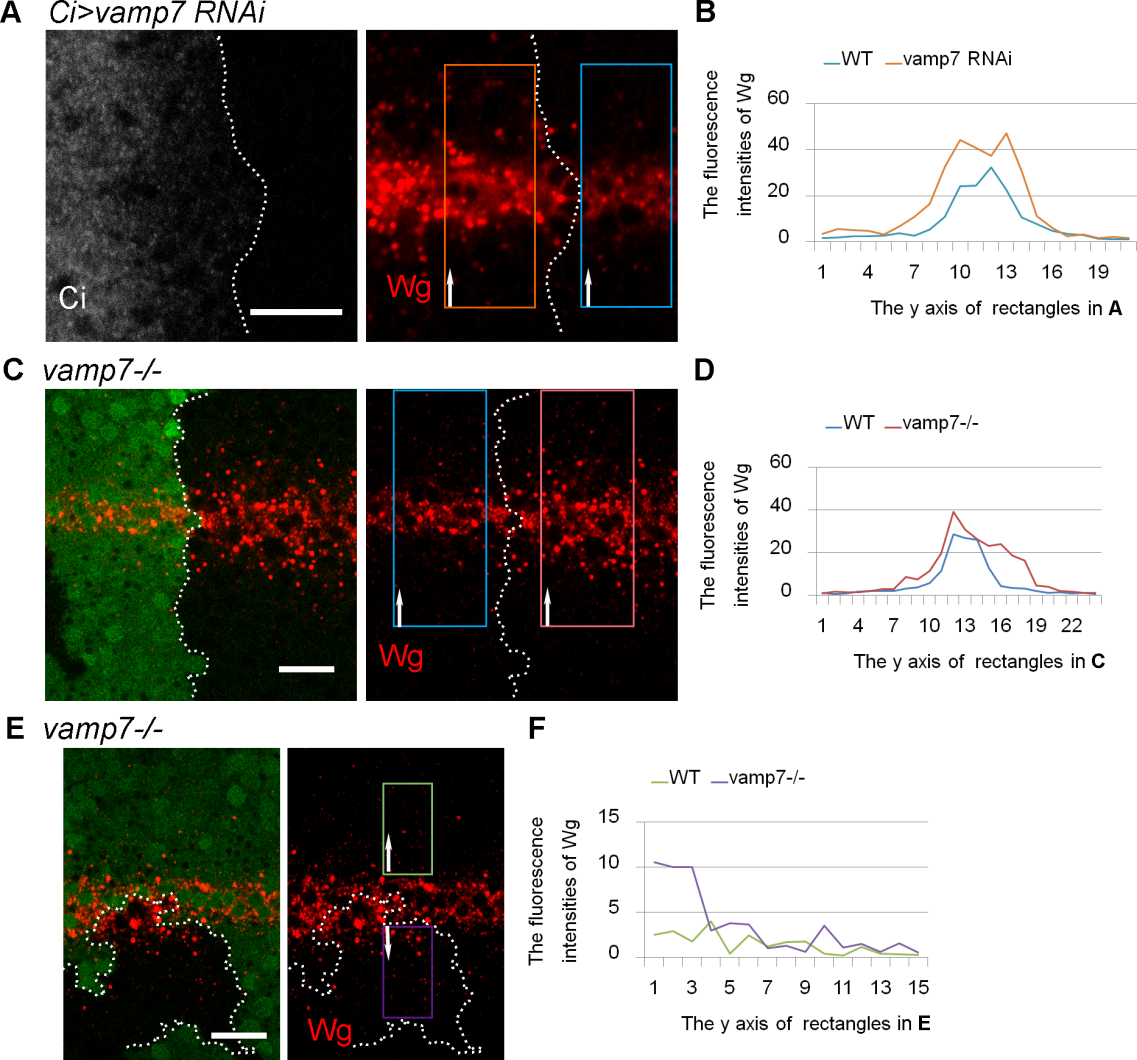
Wg levels increased in *vamp7* mutant cells. (A-E) Wg is expressed along the D/V boundary in the wing discs of drosophila late third instar larvae. (A) RNAi against *vamp7* is expressed by *ci*^*Gal4*^ driver in the anterior compartment marked by Cubitus interruptus (Ci) staining. To compare the Wg intensities, we choose two parallel rectangular areas in the anterior and posterior compartments, respectively, with their centers localizing on the intersection of the D/V axes. The Wg fluorescence intensities are shown in (B). The rectangles are divided into 21 parallel units along the direction of the arrows, and the average fluorescence intensity in each unit is measured. So do all subsequent figures. (C) The *vamp7* mutant clone is indicated by the absence of GFP and outlined by dashed lines. The Wg intensities of WT and mutant clone are shown in (D). (E) The Wg distribution in *vamp7*^-/-^ receiving cells is examined in a mutant clone close to WT producing cells, and its intensity is compared with WT receiving cells localized on the contralateral side of the D/V axis (F). Scale bars: 20 μm.

In the third instar wing disc, Wg is secreted from the producing cells at the D/V (Dorsal ventral) boundary, diffusing away on either side to form a concentration gradient. To identify novel transmembrane proteins required for Wg secretion and diffusion, we performed an RNAi screen using the *ci*^*Gal4*^ driver, which is expressed from the early stages of the embryo and continues through the whole process of imaginal discs in anterior compartments. We then compared the Wg gradient in the anterior and posterior compartments of third instar discs. Based on this screen, we found that knocking down *vamp7* led to dramatic increases in the size and the number of Wg puncta compared to those in wild-type tissue in both producing and receiving cells (Fig 1A and 1B). The somatic clone analysis utilizing a *vamp7* mutant allele confirmed the specificity of the phenotype (Fig 1C and 1D). The quantitative analysis of the Wg fluorescence intensities show that Wg puncta can spread in a little bit far away from the producing cells in *vamp7* mutant background. We further tried to investigate the effect of *vamp7* on the Wg distribution in receiving cells by generating mutant clones close to wild-type producing cells (Fig 1E and 1F). We found that Wg level still increased in the mutant receiving cells, which suggest that the effect of Vamp7 on the receiving cells was independent of that on the producing cells. Moreover, the expression of a *wg-LacZ* enhancer trap was not affected by *vamp7* knockdown (S1 File), indicating that the increase in Wg levels was not the result of transcriptional alteration under these experimental conditions. Accordingly, Wg is expected to be transported unconventionally.

**Fig 2:**
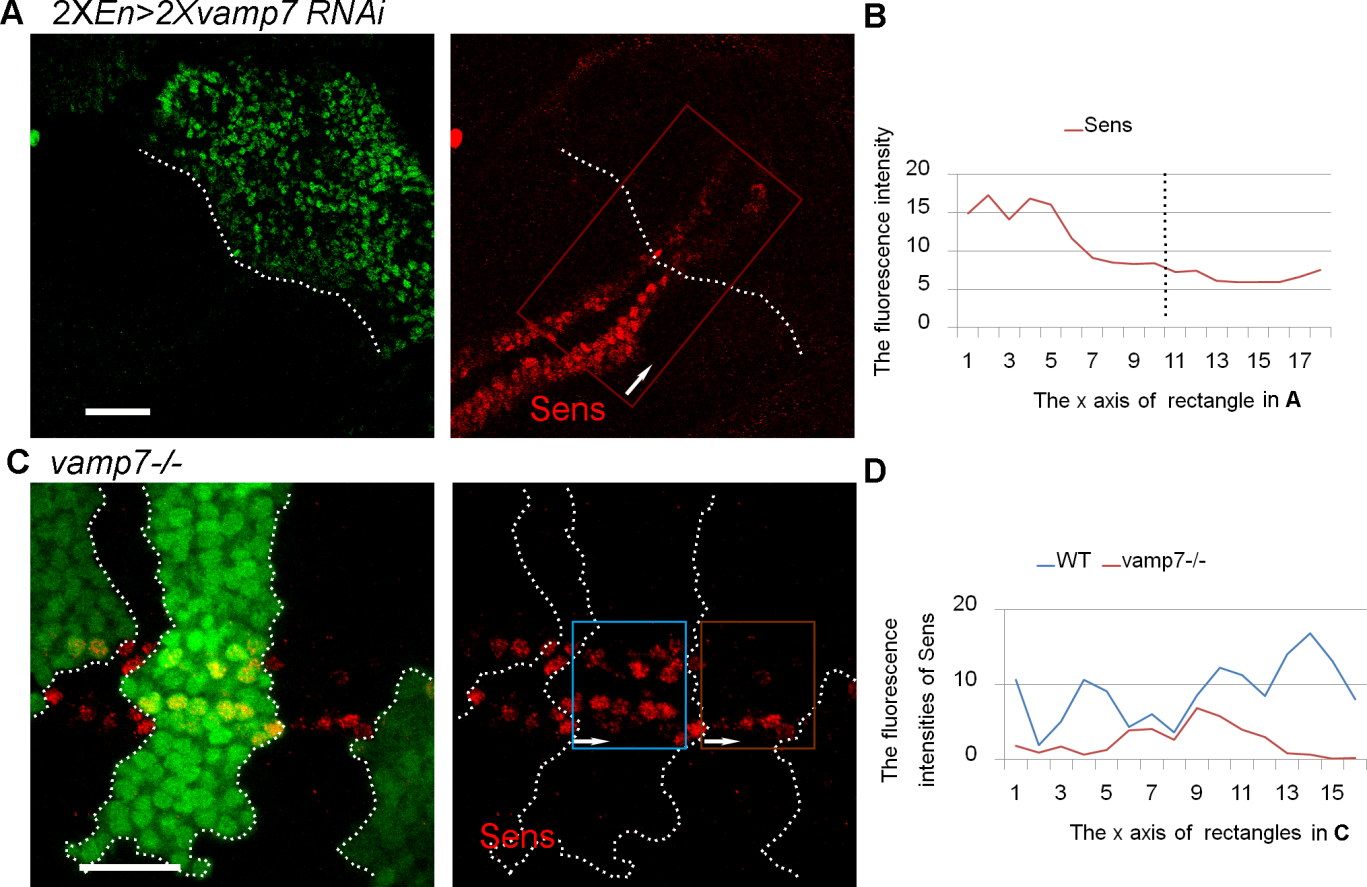
Wg signaling target gene *sens* decreases in *vamp7* mutant cells. (A) Two copies of *en*^*Gal4*^ are utilized to drive double *vamp7*^*RNAi*^. The posterior compartment is identified by En staining. (B) Sens intensity is measured in the rectangular area along the D/V axis. (C) The absence of GFP indicates the mutant clones, Sens intensities in the rectangular areas are shown in (D). Sens levels reduced in the large mutant clones straddling the D/V boundary. Scale bars: 20 μm. The intensity data are collected along the direction of arrows in the figure.

To investigate the effect of VAMP7 on Wg signaling, we examined the expression of *Sense* (*Sens*), a high-threshold Wg target gene. When two copies of *vamp7* RNAi were expressed by the *en*^*Gal4*^ driver, a reduction of Sens was observed in the posterior compartment (Fig 2A and 2B). We further generated a large mutant clone along the D/V boundary and observed a similar reduction of Sens in *vamp7* mutant cells (Fig 2C and 2D). These data suggested that the *vamp7* mutant affected Wg signaling.

### VAMP7 is required for proper Dlp levels in the region of the D/V boundary

**Fig 3:**
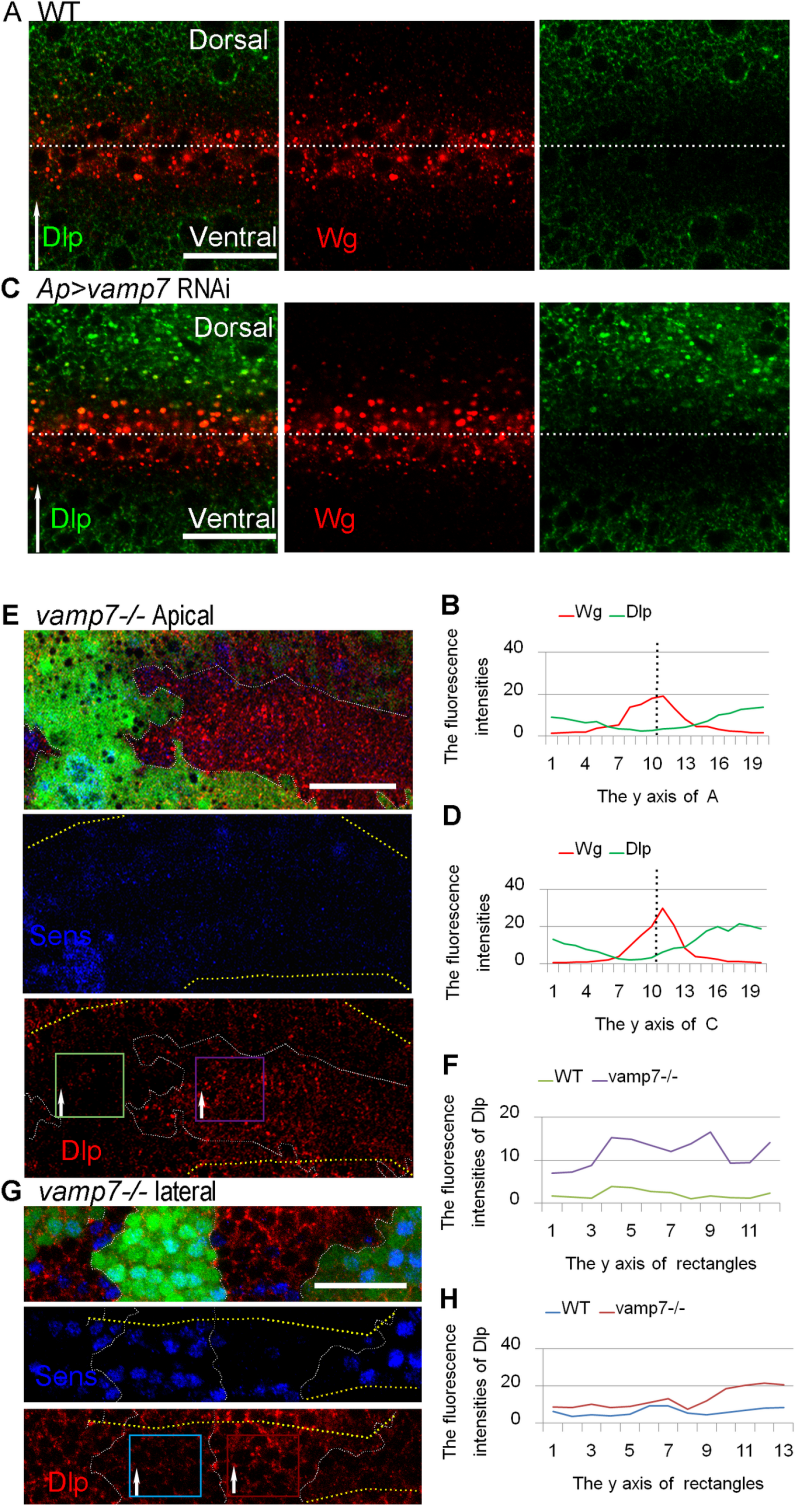
Dlp levels increase in *vamp7* mutant cells along the D/V boundary. (A, C) Staining of Wg and Dlp are carried out in WT and a*p*^*Gal4*^-driven *vamp7* RNAi wing discs. All wing discs are oriented dorsal top. (B, D) The fluorescence intensities of Wg and Dlp are measured and the intensity plots are shown in (B) and (D), respectively. (E and G) Clones of *vamp7*^*-/-*^ mutant cells are designated by the absence of GFP. Dlp and Sens are stained in discs bearing *vamp7*^*-/-*^ mutant clones, and the D/V boundary is marked with Sens. Section of (E) is taken at the apical region, and section of (G) is taken a little further down. Rectangles from the mutant clone and WT compartment are taken, and Dlp intensities are measured in the rectangular regions (F and H). Dlp levels are slightly increased in the *vamp7*^*-/-*^ mutant cells compared with the wild-type cells along the D/V boundary. Scale bars: 20 μm.

As a membrane-associated protein, Dlp is found throughout the wing imaginal disc, although presenting a reduced level in the apical region of Wg-producing and nearby Wg-receiving cells in third instar larvae [47]. The well-observed inverse expression between Wg and Dlp was shown in Fig 3A and 3B. Interestingly, we found that Dlp increased in the apical region of the D/V boundary when *vamp7 RNAi* were driven by the *Ap*^*Gal4*^ driver (Fig 3C and 3D). Next, we generated *vamp7*^*-/-*^ mutant clones across the D/V boundary and analyzed Dlp distribution in the Wg high-level region characterized by staining of the Wg signaling target gene *Sens* (Fig 3E–3H). In addition, we found that Dlp increased in the both apical region and lateral side in the mutant cells (Fig 3F and 3H), consistent with the results in *vamp7*-defective cells driven by *ap*^*Gal4*^. Our data suggest that Dlp level is enhanced by *vamp7* reduction.

### Dlp might encounter the accumulated Wg in Rab4-dependent recycling endosomes in the *vamp7* mutant cells

**Fig 4:**
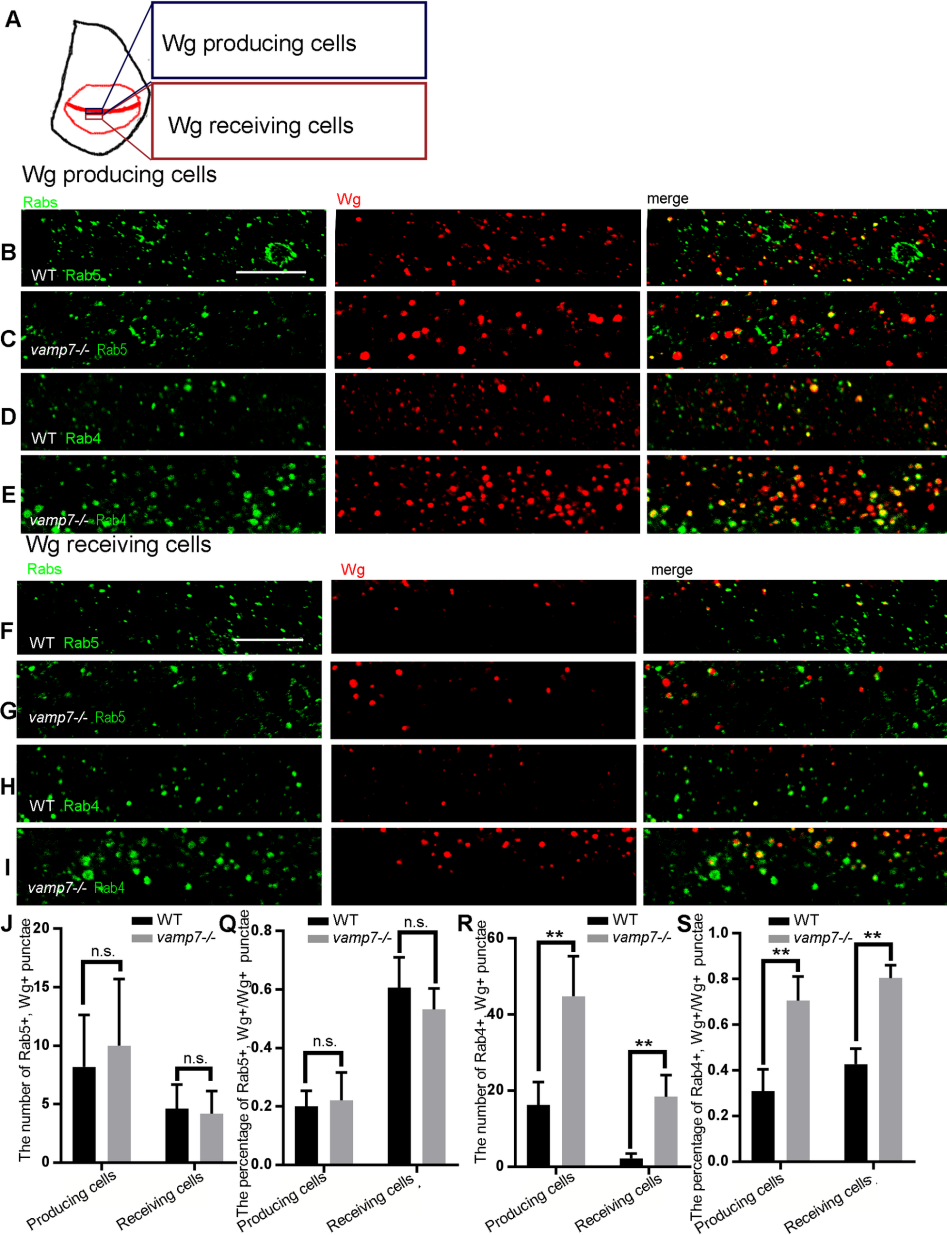
Wg accumulates in the Rab4-dependent recycling endosomes in *vamp7* mutant receiving cells. The pictures of producing cells and receiving cells are taken from the position shown in A. (B-I) Endosomes are marked by Rab5 or Rab4 in WT and *vamp7*^*-/-*^ receiving cells, respectively. Confocal sections are taken from 3 μm below the apical surface of the epithelium. Scale bars: 10 μm. We counted the number of Rab4 and Wg double positive puncta and calculated the percentage of them in total Wg positive puncta in the region of five different wing discs, and conducted a T test analysis (J) The number of Rab5 positive Wg puncta. (Q) Summary of and the percentage of Rab5 and Wg double positive puncta in the total Wg positive puncta. (R) The number of Rab4 positive Wg puncta. (S)The percentage of Rab4 and Wg double positive puncta among the total Wg positive puncta. In this and all subsequent figures, n.s., not statistically significant, *, p<0.01, **, p<0.001.

We observed that when Wg-producing cells were WT, Wg still increased in the neighboring *vamp7*^***-/-***^ mutant receiving cells. Since Vamp7 is a SNARE protein that mediates endosome fusion, and Wg is actively endocytosed in the wing disc [48], we wonder if Wg endocytosis is affected in *vamp7*^***-/-***^ mutant cells. Rabs mediate early endosome sorting, while Rab4 and Rab11 regulate cargo recycling from early endosomes to the PM and MVBs, respectively [49]. Rab7 is believed to appear in the late endosomes. In addition, LAMP1 is used to mark lysosomes. We observed that most of the Wg was present in Rab5- or Rab4-positive puncta (Fig 4), and few Wg puncta were Rab11- or LAMP1-positive (S2 File). Moreover, we found that Rab7 positive puncta were swollen (S3 File), so we could not determine whether Rab7 still marked MVBs. Next, we quantitatively analyzed the Wg in the early endosomes and the rapid recycling endosomes (Fig 4J–4S). We found that both the number and the proportion of Rab4 Wg double positive puncta in total Wg puncta were increased in Wg-producing cells under *vamp7*^***-/-***^ mutant background (Fig 4R and 4S), while, that of Rab5 positive ones were no significant difference (Fig 4J and 4Q). The similar phenomena occurred in Wg-producing cells. Overall, most Wg accumulated in the Rab4-dependent recycling endosomes in *vamp7*^***-/-***^ mutant background.

Previous studies have suggested that Dlp can promote Wg spreading [32, 47]. Consistent with that, we observed that knocking down of Dlp lead to the defect of Wg spreading (S4 File). It is also reported that Dlp undergo transcytosis from the apical surface to the basolateral membrane [50], so does Wg [22]. Further, overexpression experiments have shown that Dlp could interact with Wingless during its transcytosis [50]. Therefore, a question comes out: whether Dlp and Wg could encounter each other during endocytosis?

**Fig 5:**
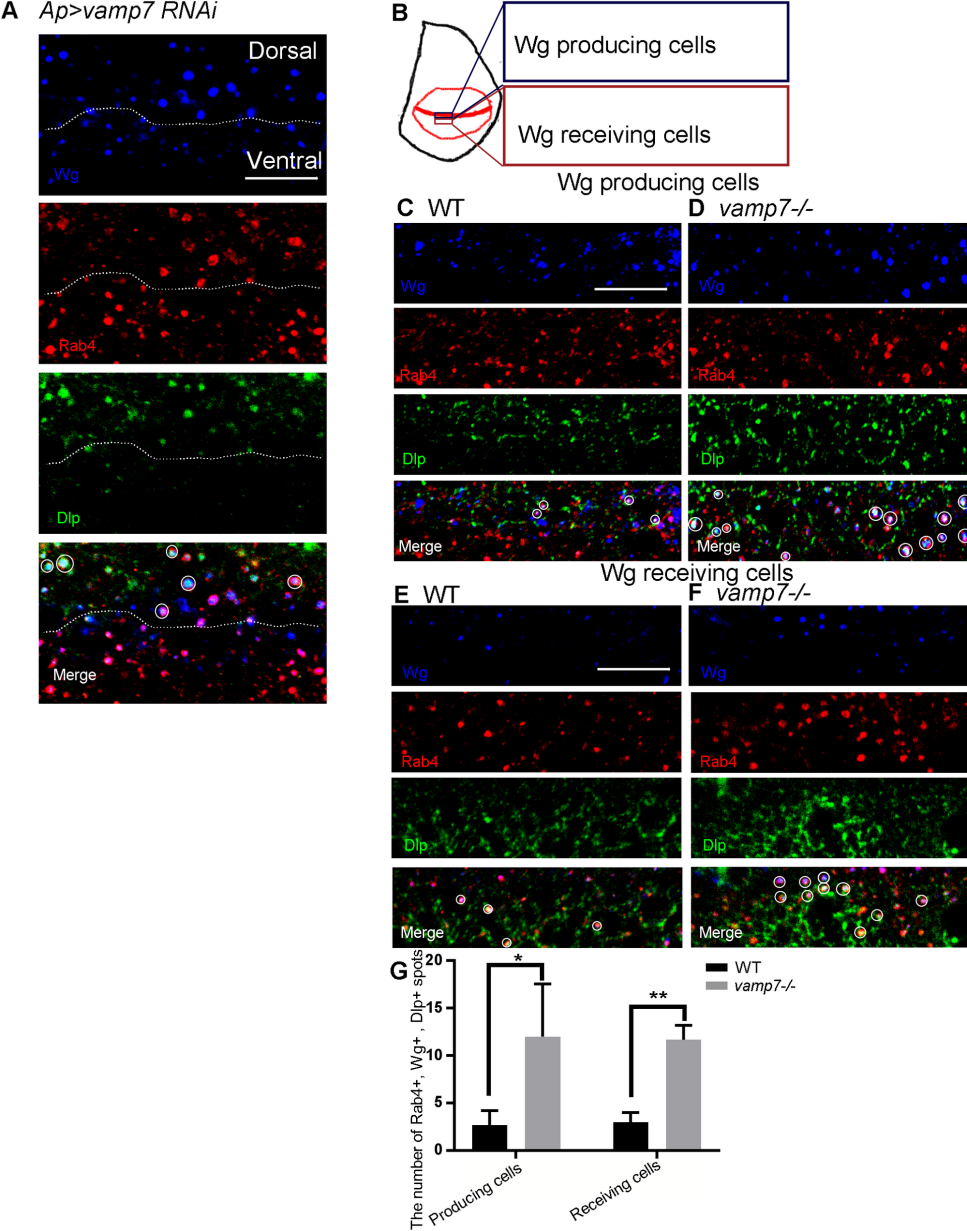
The number of Wg, Dlp and Rab4-positive puncta is increased in the *vamp7* mutant cells. (A) Knockdown of *vamp7* by *Ap*^*Gal4*^ is carried out in the dorsal compartment. The pictures of producing cells and receiving cells are taken from the position shown in B. (C-F) Wg, Dlp, and Rab4 are stained in the producing or receiving cells of WT or *vamp7*^*-/-*^ discs, respectively. Scale bars: 10 μm. The numbers of Rab4, Dlp and Wg triple positive puncta were counted in the region of five different wing discs in (C-F) are summarized in (G).

Here, we try to examine whether Wg and Dlp could exist in Rab4 recycling endosomes simultaneously. Firstly, we found that Wg, Dlp and Rab4 triple positive puncta dramatically increased when *vamp7* was knock-down in the dorsal compartment by *Ap*^*Gal4*^ driver (Fig 5A), the number of Wg, Dlp and Rab4-positive puncta also increased significantly in both *vamp7*^*-/-*^ mutant producing cells and receiving cells (Fig 5C–5G). The co-localization of Wg and Dlp implies that they do have opportunity to meet each other in the same intracellular organelle. Furthermore, the increased number of Wg, Dlp and Rab4-positive puncta indicates that *vamp7* mutation improves the exposure of Wg to Dlp.

## Discussion

### The role of VAMP7 in Wg intracellular trafficking

There are two models describing how the apically secreted Wg encounters basolateral receptors at receiving cells. One suggests that Wg and receptors can be internalized separately, and then, endosome fusion results in Wg and receptor interaction in the receiving cells [51]. Another model proposes that apically secreted Wg undergoes endocytosis and will be transported to the basolateral surface in the producing cells [22], then spread to the receiving cells for the interaction with receptors. Therefore, Wg is actively endocytosed in both receiving cells and producing cells.

In this study, we found that Wg distribution was affected in both receiving and producing cells in *vamp7*^*-/-*^ mutant background (Fig 1). Further investigation indicated that Wg double labeled puncta significantly increased, so did the percentage of Rab4 and Wg double staining puncta (Fig 4). Thus, we suggested that VAMP7 is required for Wg endocytosis in the both receiving cells and producing cells in *Drosophila* wing disc, and its mutation leads to Wg accumulating in endocytic organelles but not degradation (S2 File). Rab4 dependent recycling endosomes can recruit proteins from the early endocytic organelles, which may finally lead to increased level of Wg in Rab4 dependent recycling endosomes.

### Effect of Vamp7 mutation on Wg signaling

Although endocytosis has been demonstrated for Wg transport, there is still debate about whether endocytosis plays a direct role in the Wg signaling. Classically, the early step of endocytosis is thought to contribute positively to signaling, as early endosomes can recruit signaling components [52], while subsequent vesicle transport may downregulate signaling by sequestrating signaling components in endosomes or degradating them in lysosomes [53]. Here, we found that the expression of the Wg target gene *sens* was reduced in *vamp7* mutant cells (Fig 2). One possibility is that Rab4 recycling endosomes may recruit Wg from early endosomes. As a previous report found that the expression of activated forms of Rab4 suppressed the ability of Rab5 to enhance activation of Wg pathway [54], Wg accumulation in Rab4 recycling endosomes may affect Wg signaling. Another possible reason is that *vamp7* mutation enhances the level of Wg signaling inhibitors.

### The role of Dlp in modulating Wg signaling

Dlp is a membrane-associated glypican that can interact with Wg by its core protein on the cell surface [32], and suppresses Wg target gene *sens* [33]. However, the functional significance of interaction between Wg and Dlp inside the cell has not been well elucidated. In this study, we showed that Wg might encounter endogenous Dlp in Rab4 dependent recycling endosomes, and *vamp7* mutation could improve the levels of Dlp and Wg in Rab4 dependent recycling endosomes (Fig 5). Previous studies proposed that Dlp competes with Wg receptors to interact with Wg, and the signaling activity may be determined by the relative levels of receptor and Dlp. We suggested that competition between Dlp and receptors might not only occur on the cell surface but may have started from intracellular vesicles. The increased levels of Dlp and Wg in Rab4 dependent recycling endosomes may lead to Sens reduction.

In conclusion, we showed that an endocytic pathway involving VAMP7 regulates Wg and Dlp trafficking. This route adds another layer of spatial regulation in the Wg signaling pathway. Additional work will be needed to determine the functional significance of this route in other *Drosophila* tissues and whether *vamp7* is required for vertebrate Wnt trafficking.

## Supporting information

S1 File Wg-LacZ expression is unaffected in *vamp7* mutant cells. *En*^*Gal4*^ is used to drive *vamp7*^*RNAi*^. The posterior compartment is characterized by Engrailed staining. Scale bars: 20 μm.
(TIF)

S2 File Wg accumulates in the Rab4-dependent recycling endosomes in *vamp7* mutant producing cells. The pictures are taken from the position shown in A. (B, C, F and G) Endosomes are marked by LAMP1 in WT and *vamp7*^*-/-*^ receiving cells (D, E, H and I) *UAS-Rab 11-GFP* is overexpressed by *ci*^*Gal4*^ in wild-type and *vamp7*^*-/-*^ discs. Confocal sections are taken from 3 μm below the apical surface of the epithelium. Scale bars: 10 μm.
(TIF)

S3 File Rab7-marked vesicles are swollen in *vamp7* mutant cells. (A) *Ap*^*Gal4*^ is used to drive *UAS-Rab 7-GFP*. (B) *Vamp7*^*RNAi*^ and *UAS-Rab 7-GFP* are expressed by *Ap*^*Gal4*^ driver. The image is taken from the dorsal compartment in the wing disc. Scale bars: 10 μm.
(TIF)

S4 File Dlp favors Wg transport in *vamp7* mutant cells. RNAi against *dlp* is used in wild-type discs (A) and *vamp7*^*-/****-***^ discs (C). Wg intensities are shown in (B and D). Scale bars: 20 μm.
(TIF)
