# Supplementary figures and images for: Drosophila VAMP7 regulates Wingless intracellular trafficking
Source: PLoS One. 2017 Oct 24;12(10):e0186938. doi: 10.1371/journal.pone.0186938 (PMC5655445; doi:10.1371/journal.pone.0186938)

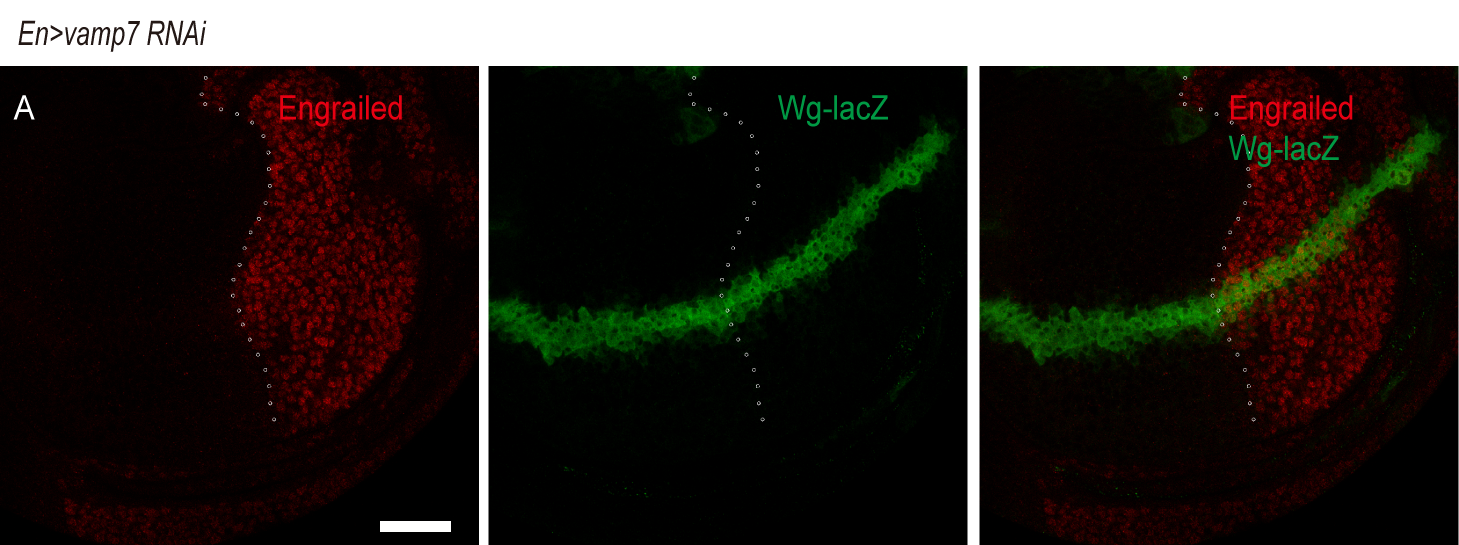

Supplement: S1 File — EnGal4 is used to drive vamp7RNAi. The posterior compartment is characterized by Engrailed staining. Scale bars: 20 μm. (TIF) [file pone.0186938.s001.tif]

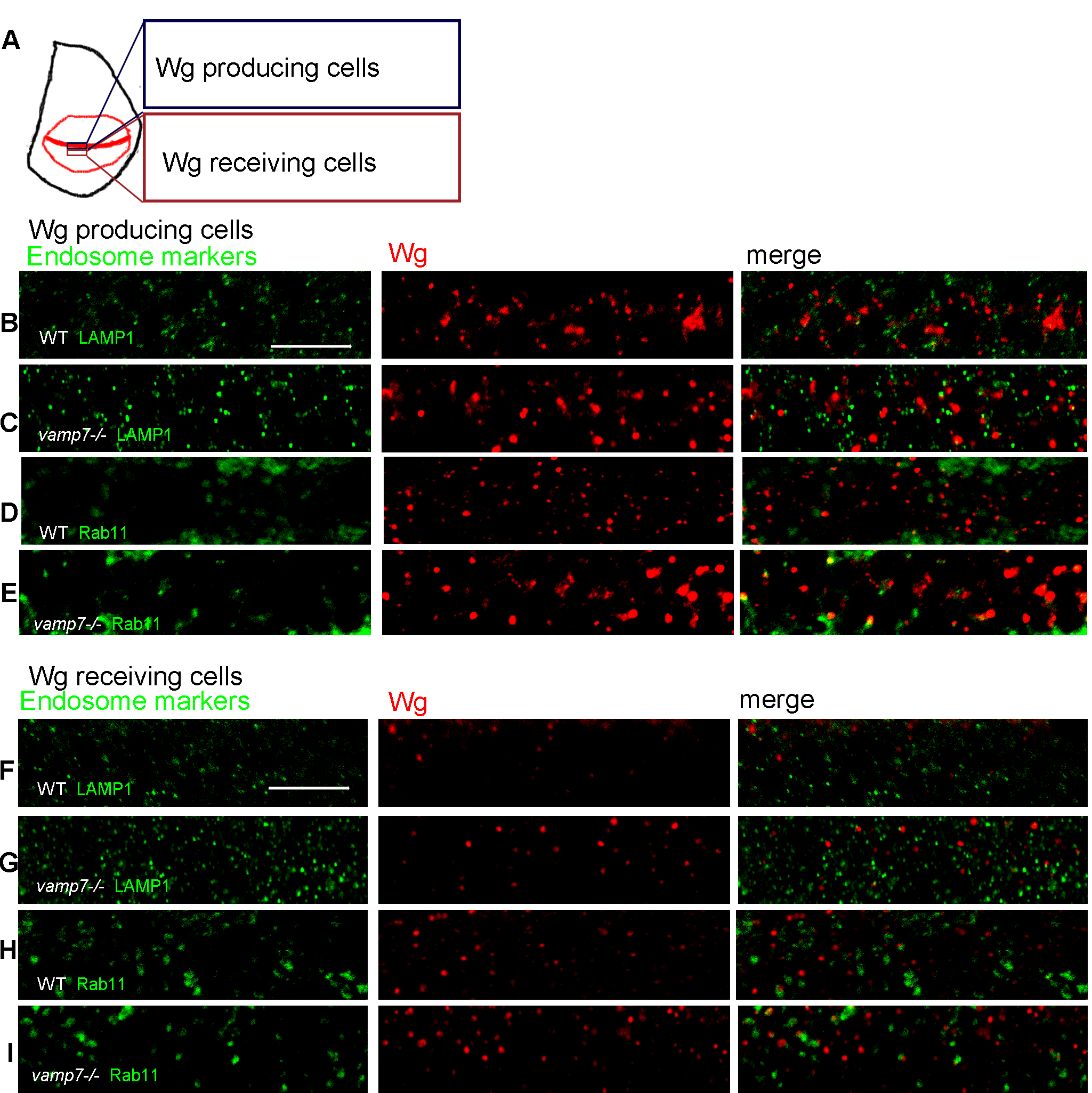

Supplement: S2 File — The pictures are taken from the position shown in A. (B, C, F and G) Endosomes are marked by LAMP1 in WT and vamp7-/- receiving cells (D, E, H and I) UAS-Rab 11-GFP is overexpressed by ciGal4 in wild-type and vamp7-/- discs. Confocal sections are taken from 3 μm below the apical surface of the epithelium. Scale bars: 10 μm. (TIF) [file pone.0186938.s002.tif]

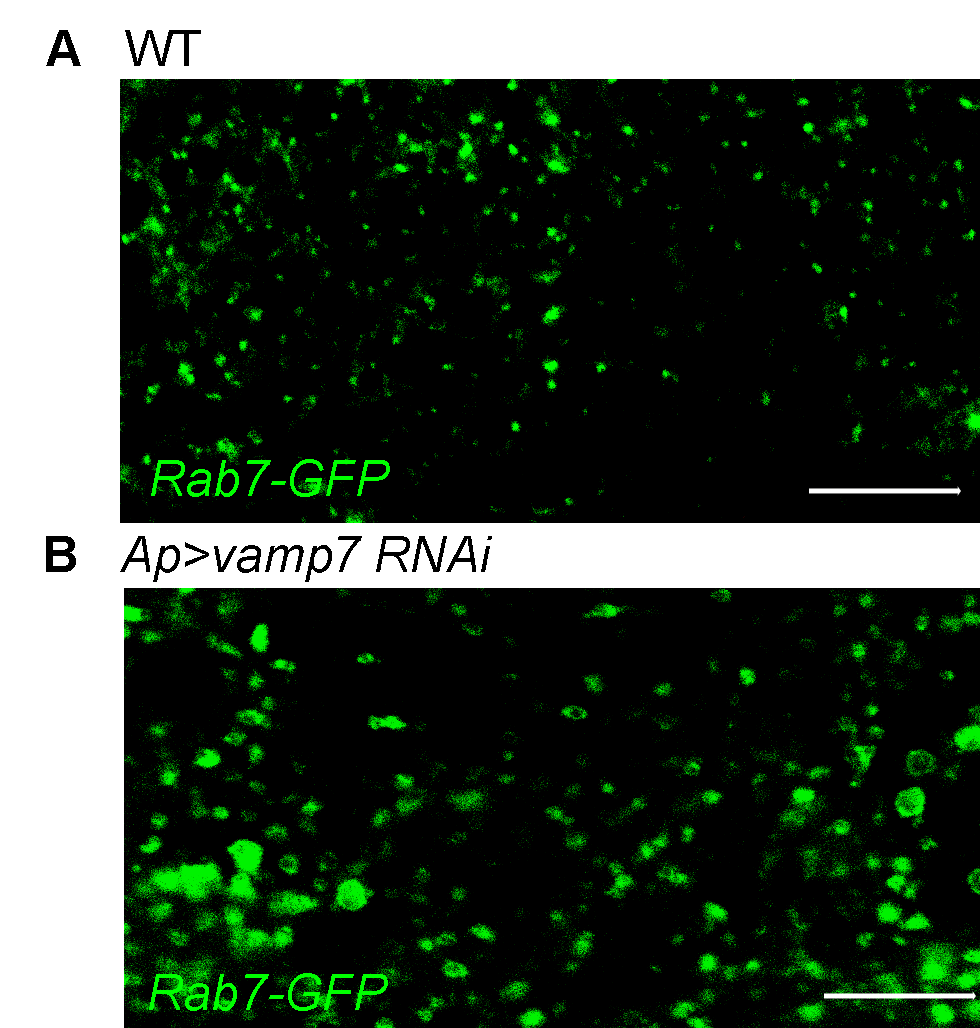

Supplement: S3 File — (A) ApGal4 is used to drive UAS-Rab 7-GFP. (B) Vamp7RNAi and UAS-Rab 7-GFP are expressed by ApGal4 driver. The image is taken from the dorsal compartment in the wing disc. Scale bars: 10 μm. (TIF) [file pone.0186938.s003.tif]

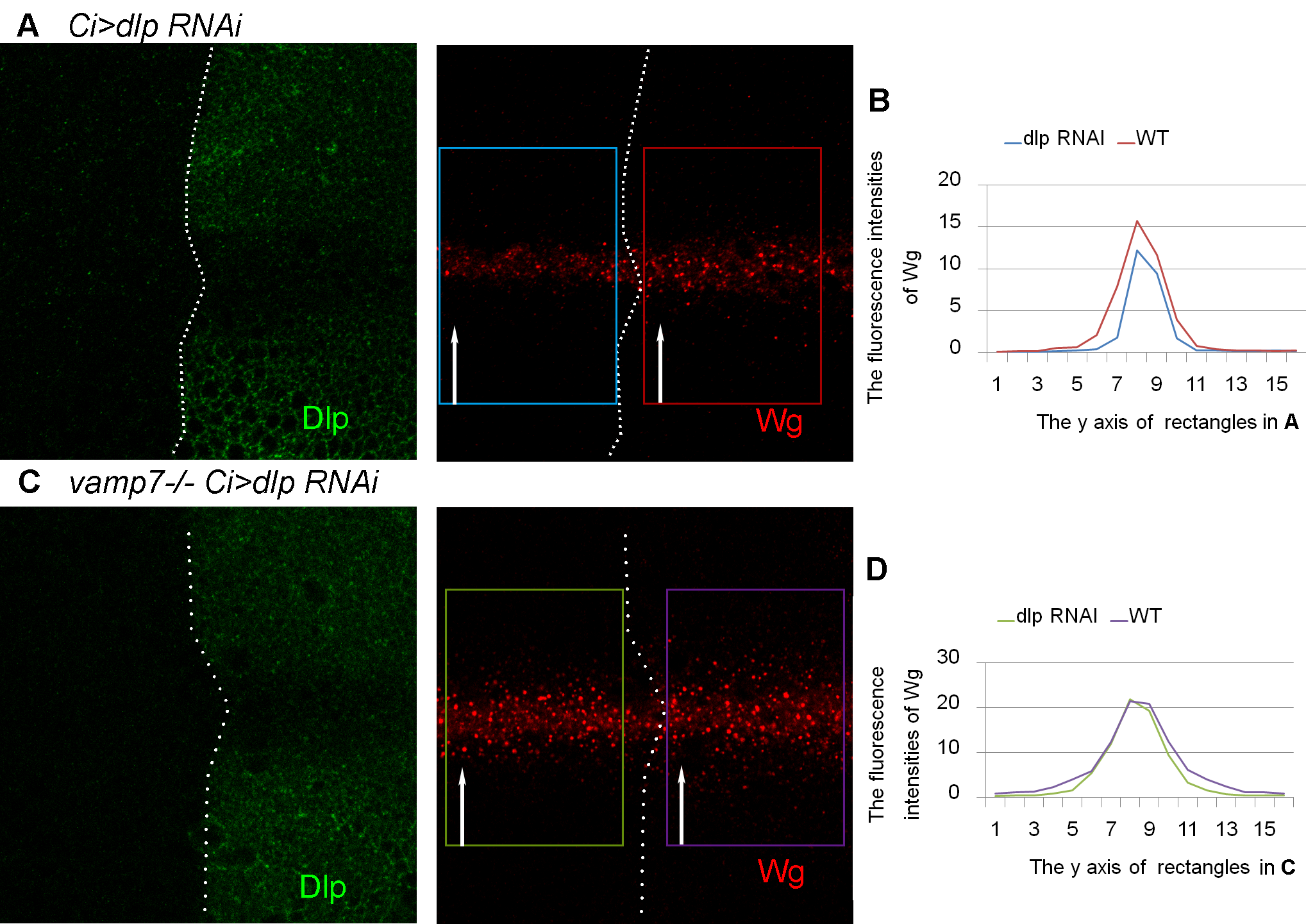

Supplement: S4 File — RNAi against dlp is used in wild-type discs (A) and vamp7-/- discs (C). Wg intensities are shown in (B and D). Scale bars: 20 μm. (TIF) [file pone.0186938.s004.tif]
